# Food Insecurity and Cardiovascular Disease Risk Factors among Mississippi Adults

**DOI:** 10.3390/ijerph15092016

**Published:** 2018-09-15

**Authors:** Vincent L. Mendy, Rodolfo Vargas, Gerri Cannon-Smith, Marinelle Payton, Byambaa Enkhmaa, Lei Zhang

**Affiliations:** 1 Department of Epidemiology and Biostatistics, School of Public Health, Jackson State University, Jackson, MS 39213, USA; marinelle.payton@jsums.edu; 2 Office of Health Data and Research, Mississippi State Department of Health, Jackson, MS 39216, USA; rodolfo.vargas@msdh.ms.gov (R.V.); gerricannonsmith@bellsouth.net (G.C.-S.); lei.zhang@msdh.ms.gov (L.Z.); 3 Center of Excellence in Minority Health and Health Disparities, Institute of Epidemiology and Health Services Research, School of Public Health, Jackson State University, Jackson, MS 39213, USA; 4 Department of Internal Medicine, School of Medicine, University of California Davis, Davis, CA 95616, USA; ebyambaa@ucdavis.edu

**Keywords:** cardiovascular disease, risk factors, food insecurity, Behavioral Risk Factor Surveillance System (BRFSS), Mississippi

## Abstract

*Background*: Food insecurity is a public health problem. There is limited data on food insecurity in Mississippi. *Methods*: We analyzed data from the 2015 Mississippi Behavioral Risk Factor Surveillance System, which included the Social Context Module for 5870 respondents. Respondents who indicated that in the past 12 months they were “always”, “usually”, or “sometimes” “worried or stressed about having enough money to buy nutritious meals” were considered food insecure. Food insecurity was compared across sociodemographic and health characteristics using chi-square tests, and the association between food insecurity and select cardiovascular disease risk factors was assessed using logistic regression. *Results*: The prevalence of food insecurity was 42.9%. Compared to the referent group, Mississippi adults with high blood pressure had 51% higher odds, those with diabetes had 30% higher odds, those who were not physically active had 36% higher odds, and those who consumed fewer than five fruits and vegetables daily had 50% higher odds of being food insecure. *Conclusion*: Among Mississippi adults, food insecurity is associated with high blood pressure, diabetes, obesity, fruit and vegetable consumption, physical inactivity, and smoking.

## 1. Introduction

Food insecurity, which is defined by the United States Department of Agriculture (USDA) as “a household-level economic and social condition of limited or uncertain access to adequate food” [1], is a public health problem [2]. In 2015, 15.8 million U.S. households (12.7%) were food insecure [3]. Food insecurity is associated with poor diet [4], and is linked to a myriad of negative health outcomes including heart disease, high blood pressure, diabetes, obesity, poorer general health, increased health-care utilization, depression [5,6,7,8,9,10], and cardiovascular health [11,12]. In addition, food insecurity is associated with poor control of high blood pressure [13].

Cardiovascular disease (CVD) (i.e., coronary heart disease, myocardial infarction, heart failure, angina, and stroke) is another significant public health problem, especially in Mississippi, where the condition is the leading cause of death. In 2015, the state’s CVD death rate was 1.4 times higher than the national rate [14]. Further, the prevalence of CVD risk factors such as high blood pressure, diabetes and obesity is disproportionately higher in Mississippi than in the nation as a whole [15]. The Mississippi State Department of Health (MSDH), in collaboration with the Centers for Disease Control and Prevention (CDC) and local stakeholders, is currently implementing programs—through the Mississippi Delta Health Collaborative (MDHC)—that seek to address CVD by increasing access to healthy food and high blood pressure prevention and control in the 18-county Mississippi Delta region (www.healthyms.com/MDHC).

There is limited data on both food insecurity and CVD risk in Mississippi. In 2015, the Behavioral Risk Factor Surveillance System (BRFSS) Social Context Module measured food insecurity for the first time, via a question asking about stress associated with the affordability of nutritious meals. We used the responses to examine the association between select CVD risk factors and food insecurity among Mississippi adults, and to assess differences in food insecurity by sociodemographic and health characteristics.

## 2. Methods

We analyzed data from the 2015 Mississippi BRFSS, which included the Social Context Module. Current analysis has been restricted to respondents who self-identified as black or white; these two racial groups accounted for 96.6% of the study population. The BRFSS is a state-based, random-digit-dialed telephone survey of the U.S. noninstitutionalized civilian population aged 18 years or older. The survey was conducted in all 50 states, the District of Columbia and three U.S. territories (Puerto Rico, Guam and the U.S. Virgin Islands). Data from the BRFSS produced reliable and valid assessments of health risk factors [16]. Post-stratification weights were used to adjust for nonresponse, noncoverage, and disproportionate selection of populations [8,16]. The BRFSS was approved by the human research review board at each state’s department of health. Detailed information about BRFSS is available at www.cdc.gov/brfss/. This study was deemed exempt by the Mississippi State Department of Health Institutional Review Board.

### 2.1. Food Insecurity

Food insecurity was defined based on the following question: “How often in the past 12 months would you say you were worried or stressed about having enough money to buy nutritious meals?” Respondents who answered “always,” “usually” or “sometimes” were considered food insecure, and those who responded “rarely” or “never” were considered food secure [8].

### 2.2. CVD Risk Factors

High blood pressure was defined as a “yes” response to the question, “Have you ever been told by a doctor, nurse or other health professional that you have a high blood pressure?” High cholesterol was defined as a “yes” response to the question, “Have you ever been told by a doctor, nurse or other health professional that your blood cholesterol is high?” Diabetes was defined as a “yes” response to the question, “Have you ever been told by a doctor that you have diabetes?” Current smoking was defined as having smoked at least 100 cigarettes during the respondent’s lifetime and currently smoking at the time of the survey. Body mass index (BMI) (calculated with self-reported height and weight) was classified into three categories (normal weight, BMI < 25.0; overweight, BMI 25.0 to <30.0; and obese, BMI ≥ 30.0). Physical inactivity was defined as a “no” response to the question “During the past month, other than your regular job, did you participate in any physical activities or exercise such as running, calisthenics, golf, gardening, or walking for exercise?” Binge drinking was defined as having five or more drinks on one occasion for males, and as having four or more drinks on one occasion for females. Fruit and vegetable consumption was based on the total number of fruits and vegetables respondents reported consuming per day. Responses were categorized as either less than five fruits/vegetables, or five or more fruits/vegetables consumed per day.

### 2.3. Statistical Analyses 

Weighted prevalence and 95% confidence intervals (CI) were calculated. Food insecurity was compared across sociodemographic characteristics using chi-square tests, and the associations between food insecurity and select CVD risk factors were assessed using logistic regression models including controls for age, gender, race, education, annual household income, and health insurance. SAS version 9.4 (SAS Institute, Cary, NC, USA) was used to perform all statistical analyses, which accounted for the complex sample design; significance levels were determined based on a *p*-value less than 0.05.

## 3. Results

The analyses were based on data from 5870 Mississippi adults. The mean age of respondents was 47.4 years; 38.4% were black, 52.6% were women, just over half (51.7%) had greater than a high school education, 56.8% were employed, and about a quarter (24.9%) had an annual household income of less than $20,000 (Table 1). 

The overall prevalence of food insecurity was 42.9% (95% CI, 40.7–45.0). The prevalence of food insecurity differed for several background characteristics: blacks (53.7%, 95% CI, 50.0–57.3, *p* < 0.0001) had a higher prevalence than whites, females (46.2%, 95% CI, 43.5–48.9, *p* = 0.0011) had a higher prevalence than males, those with high blood pressure (45.8%, 95% CI, 42.8–48.7, *p* = 0.0163) had a higher prevalence than those without high blood pressure, and those without health insurance (65.0%, 95% CI, 59.3–70.8, *p* < 0.0001) had a higher prevalence than those with health insurance. In addition, there were significant differences in the prevalence of food insecurity by age group, educational level, employment status, marital status, annual household income, body mass index, and smoking status (*p* < 0.0001) (Table 2).

The results of the regression models including controls for age, gender, race, education, annual household income, and health insurance also showed associations between food insecurity and sociodemographic and health characteristics. Mississippi adults with high blood pressure had 51% higher odds (adjusted odds ratio (AOR) 1.51, 95% CI, 1.21–1.88, *p* = 0.00082) than those without high blood pressure, those with diabetes had 30% higher odds (AOR 1.30, 95% CI, 1.02–1.65, *p* = 0.0365) than those without diabetes, those who were not physically active had 36% higher odds (AOR 1.36, 95% CI, 1.10–1.68, *p* = 0.0043) than those who were physically active, and those who consumed fewer than five fruits and vegetables daily had 50% higher odds (AOR 1.50, 95% CI, 1.05–2.45, *p* = 0.0259) of being food insecure than those who consumed five or more fruits and vegetables daily (Table 3). Similarly, relative to those with a BMI < 25.0, the odds of being food insecure were 68% higher (AOR 1.68, 95% CI, 1.28–2.21, *p* = 0.0002) among those with a BMI of ≥30.0, and 38% higher (AOR 1.38, 95% CI, 1.05–1.81, *p* = 0.0227) among those with a BMI of 25.0–29.9. Finally, current smokers had 82% higher odds of being food insecure (AOR 1.82, 95% CI 1.40–2.37, *p* < 0.0001) than nonsmokers (Table 3).

## 4. Discussion

In 2015, the prevalence of food insecurity among Mississippi adults was three times the U.S. average (12.7%) [1]. An estimated two out of every five (*n* = 742,381 (42.9%)) Mississippi adults were food insecure. The prevalence of food insecurity was significantly higher among blacks than whites, and among females than males. In addition, both those with high blood pressure and those without health insurance had a higher prevalence than their counterparts. We also found significant differences in the prevalence of food insecurity by age group, educational level, employment status, marital status, annual household income, body mass index, and smoking status. When age, gender, race, education, annual household income, and health insurance were controlled, food insecurity was significantly associated with high blood pressure, diabetes, obesity, fruit and vegetable consumption, physical inactivity, and smoking status among Mississippi adults. 

The significant associations between food insecurity and high blood pressure, obesity, diabetes, and physical inactivity are consistent with the findings of previous reports from other states [8,10,17,18,19]. In 2015, an estimated 955,137 (42.4%) Mississippi adults had high blood pressure, 760,144 (36.4%) were obese, 334,024 (14.7%) had diabetes, and 779,898 (36.8%) were physically inactive [20]. In addition, high blood pressure, diabetes and obesity disproportionately affected black Mississippians (37.0% of the total population). A possible explanation for the association between food insecurity and physical inactivity is that food insecurity might lead to distress or poor health, any of which could lead to a lower level of physical activity [19].

Prior studies have shown that food insecure individuals report a higher juice intake and are less likely to engage in fat-lowering behaviors [21]. Similarly, among adults, food insecurity is adversely associated with dietary quality [22]. Mississippi adults with these chronic health conditions (high blood pressure, obesity, diabetes, and physical inactivity) could benefit from focused interventions that address the availability, accessibility and affordability of healthy food options in the state. The MSDH (Mississippi Delta Health Collaborative) and the CDC, through a cooperative agreement, are currently implementing interventions across the 18-county Mississippi Delta region (a region with a disproportionately high burden of high blood pressure and obesity). These interventions target the ABCS (aspirin for those eligible, blood pressure control, cholesterol management, and smoking cessation) of heart disease and stroke prevention. In particular, the Mayoral Health Council Initiative addresses access to healthy food (electronic benefit transfer (EBT) cards for farmers’ markets or increasing fruit/vegetable access at corner/convenience markets) and physical activity options (shared-use agreements) in the 18-county Mississippi Delta region.

Our findings are subject to the following limitations. First, the BRFSS consists of self-reported information on food insecurity and CVD risk factors, and therefore the data is subject to recall bias and social desirability bias. Second, the Mississippi BRFSS sample includes only adults (18 years of age and older); therefore, the findings may not be generalizable to children. Third, food insecurity was assessed based on a single question. Finally, because the data is cross-sectional, we cannot make causal inferences based on the results. 

## 5. Conclusions

In this study, we found that food insecurity was associated with high blood pressure, diabetes, obesity, fruit and vegetable consumption, physical inactivity, and smoking status among Mississippi adults. Programs and interventions that target food insecure individuals are needed in the state, particularity for adult Mississippians who have high blood pressure or diabetes, who are obese or physically inactive, or who currently smoke. Identifying food insecure adults and linking them to available resources in the state could play an important role in addressing disparities in high blood pressure, diabetes and obesity prevention and control in Mississippi. 

## Figures and Tables

**Table 1:** Sociodemographic characteristics of Mississippi adults, Behavioral Risk Factor Surveillance System, 2015.

Characteristic	N ^a^	% ^b^	95% CI
Age (years)			
18–34	705	30.3	28.3–32.3
35–49	935	23.0	21.4–24.6
50–64	1869	26.7	25.2–28.2
≥65	2361	20.0	18.9–21.1
Race			
Black	2099	38.4	36.5–40.2
White	3746	61.6	59.8–63.5
Sex			
Male	2052	47.4	45.5–49.3
Female	3818	52.6	50.7–54.5
Education level			
<High school graduate	787	18.4	16.8–20.0
High school or equivalent graduate	1859	29.9	28.2–31.6
>High school graduate	3207	51.7	49.8–53.6
Employment			
Employed	2184	56.8	54.8–58.8
Unemployed	631	15.0	13.4–16.6
Student	128	6.8	5.5–8.2
Retired	2067	21.4	20.1–22.7
Marital Status			
Married	2701	48.7	46.8–50.5
Widowed	1174	8.9	8.2–9.7
Divorced/separated	1041	15.5	14.2–16.7
Never married	886	27.0	25.0–28.9
Annual household income ($)			
<20,000	1485	24.9	23.2–26.5
20,000-34,999	1200	21.5	20.0–23.1
35,000-49,999	622	10.9	9.7–12.1
≥50,000	1512	27.1	25.4–28.7
Don’t know/Refused	1026	15.6	14.3–17.0
Physical Inactivity			
Yes	2175	36.6	34.8–38.5
No	3391	63.4	61.5–65.2

CI, confidence interval; ^a^ Unweighted; ^b^ Weighted percent.

**Table 2:** Food insecurity among Mississippi adults by sociodemographic and health characteristics, Behavioral Risk Factor Surveillance System, 2015.

Characteristic	Food Insecurity		*p*-Value ^b^
	% ^a^ (*n* = 1659)	95% CI	
Overall	42.9	40.7–45.0	
Age group (years)			
18–24	50.7	45.3–56.1	<0.0001
25–44	48.8	44.4–53.2	
45–64	41.5	38.2–44.9	
≥65	26.8	23.9–29.7	
Race			
Black	53.7	50.0–57.3	<0.0001
White	36.4	33.8–39.0	
Sex			
Male	38.9	35.5–42.3	0.0011
Female	46.2	43.5–48.9	
Education level			
<High school graduate	57.8	52.2–63.4	<0.0001
High school or equivalent graduate	47.7	43.8–51.5	
>High school graduate	35.6	32.8–38.3	
Employment status			
Employed	38.1	35.1–41.2	<0.0001
Unemployed	54.9	48.4–61.4	
Student	49.7	34.8–64.6	
Retired	25.3	22.0–28.6	
Marital Status			
Married	35.2	32.4–37.9	<0.0001
Widowed	34.7	29.9–39.6	
Divorced/separated	57.7	52.9–62.6	
Never married	52.2	46.6–57.8	
Annual household income ($)			
<20,000	67.6	63.6–71.6	<0.0001
20,000–34,999	49.5	45.0–54.1	
35,000–49,999	43.6	37.2–50.0	
≥50,000	18.9	15.7–22.1	
Don’t know/Refused	37.6	31.6–43.6	
Body Mass Index (BMI)			
<25.0	36.5	32.2–40.8	<0.0001
25.0–<30.0	40.1	36.5–43.7	
≥30	49.5	45.9–53.1	
Smoking status			
Current	61.1	56.5–65.8	<0.0001
Former	34.4	30.2–38.5	
Never	39.0	36.2–41.9	
Diabetes			
Yes	46.4	41.7–51.0	0.1122
No	42.2	39.8–44.5	
High blood pressure			
Yes	45.8	42.8–48.7	0.0163
No	40.5	37.5–43.6	
High Cholesterol			
Yes	40.6	37.4–43.8	0.5749
No	39.4	36.3–42.4	
Health insurance			
Yes	38.6	36.4–40.9	<0.0001
No	65.0	59.3–70.8	

CI, confidence interval; ^a^ Weighted percent; ^b^ Determined by X^2^ test.

**Table 3:** Association between food insecurity and select cardiovascular disease risk factors among Mississippi adults, Behavioral Risk Factor Surveillance System, 2015.

Characteristic	AOR ^a^	95% CI	*p*-Value
Diabetes mellitus			0.0365
Yes	1.30	1.02–1.65	
No	1.00	Referent	
High blood pressure			
Yes	1.51	1.21–1.88	0.0002
No	1.00	Referent	
High cholesterol			
Yes	1.17	0.94–1.45	0.1619
No	1.00	Referent	
Body mass index (BMI)			
≥30	1.68	1.28–2.21	0.0002
25.0–29.9	1.38	1.05–1.81	0.0227
18.5–24.9	1.00	Referent	
Smoking status			
Current	1.82	1.40–2.37	<0.0001
Former	0.97	0.75–1.25	0.8003
Never	1.00	Referent	
Binge drinking			
Yes	1.08	0.73–1.59	0.7018
No	1.00	Referent	
Physical inactivity			
Yes	1.36	1.10–1.68	0.0043
No	1.00	Referent	
Fruits/Vegetables consumed per day			
≥5	1.00	Referent	0.0259
<5	1.50	1.05–2.145	

AOR, adjusted odds ratio; CI, confidence interval; ^a^ Adjusted for age, sex, race, education, health insurance and income.
